# Post-transplant diabetes mellitus after kidney transplantation: pathogenesis, risk factors, and management strategies

**DOI:** 10.3389/fendo.2026.1829579

**Published:** 2026-04-29

**Authors:** Jianhua Long, Jiyu Zhao, Xianen Gu, Chunlei Huang

**Affiliations:** Department of Urology, Chunlei Huang Chuiyangliu Hospital Affiliated to Tsinghua University, Beijing, China

**Keywords:** GLP-1 receptor agonists, gut microbiota dysbiosis, gut–immune–metabolic axis, kidney transplantation, post-transplant diabetes mellitus, SGLT2 inhibitors

## Abstract

Post-transplant diabetes mellitus (PTDM) affects 7–39% of kidney transplant recipients and substantially worsens cardiovascular, infectious, and allograft outcomes. Although PTDM shares core pathophysiological features with type 2 diabetes—peripheral insulin resistance and impaired β-cell secretion—its etiology is fundamentally shaped by immunosuppressive therapy. Calcineurin inhibitors suppress insulin gene transcription via NFAT inhibition and exacerbate lipotoxicity; corticosteroids drive hepatic gluconeogenesis and impair GLUT4-mediated glucose uptake; and mTOR inhibitors reduce β-cell mass through mTORC1-dependent mechanisms. Chronic NF-κB/JNK-driven inflammation further amplifies insulin resistance and promotes β-cell apoptosis. Beyond these established mechanisms, we propose a unifying “gut–immune–metabolic axis” in which immunosuppression-induced gut microbiota dysbiosis—characterized by depletion of short-chain fatty acid-producing taxa (Roseburia, Faecalibacterium prausnitzii) and Akkermansia muciniphila—drives intestinal barrier dysfunction, endotoxemia, impaired FXR/TGR5-mediated GLP-1 secretion, and TMAO-associated metabolic inflammation, collectively perpetuating glucose dysregulation. Risk stratification integrates non-modifiable factors (advanced age, African American/Hispanic/South Asian ethnicity, TCF7L2 polymorphisms, autosomal dominant polycystic kidney disease) with modifiable determinants (pre-transplant dysglycemia, obesity, hypomagnesemia, hepatitis C and cytomegalovirus infections, acute rejection, and diuretic use). Diagnosis requires OGTT-centered assessment per the 2024 International Consensus guidelines, with cautious interpretation of HbA1c during the early post-transplant period. Management encompasses personalized immunosuppression (corticosteroid minimization, tacrolimus trough levels <10 ng/mL, and belatacept-based regimens in high-risk patients), structured lifestyle interventions, and emerging pharmacotherapies—particularly SGLT2 inhibitors and GLP-1 receptor agonists—which offer cardiometabolic benefits beyond glycemic control. Microbiome-targeted strategies, including prebiotics, probiotics, and fecal microbiota transplantation, represent a conceptually compelling frontier warranting prospective investigation. This framework reframes PTDM as a multi-hit, immunometabolic syndrome and provides a translational roadmap toward precision prevention and improved long-term transplant outcomes.

## 1 Introduction

Kidney transplantation (KT) is the preferred treatment for patients with end-stage renal disease (ESRD), offering superior survival and quality of life compared to dialysis (1, 2). Advances in surgical techniques and immunosuppressive regimens, especially the introduction of calcineurin inhibitors (CNIs), have significantly improved short- and medium-term graft survival (3). However, long-term outcomes remain suboptimal, in part due to metabolic complications, with post-transplant diabetes mellitus (PTDM) being a major concern (4, 5).

PTDM was first reported in the 1960s (6), with terminology evolving over time. In 2003, the term “new-onset diabetes after transplant” (NODAT) was introduced to differentiate it from pre-existing, undiagnosed diabetes (7, 8). However, a 2014 international consensus meeting recommended reverting to the term PTDM, as it more accurately reflects the timing of diagnosis rather than onset and acknowledges the practical challenges associated with pre-transplant diabetes screening (9). PTDM is linked to adverse outcomes, including increased risks of cardiovascular disease, infections, graft dysfunction, and graft loss (1, 4, 10–12). The diagnosis of PTDM is based on established glycemic thresholds as outlined in international consensus guidelines (13).

The pathophysiology of PTDM is not fully understood but shares similarities with type 2 diabetes mellitus (T2DM), such as insulin resistance and impaired insulin secretion. However, PTDM also involves unique transplant-related mechanisms (14, 15). This review aims to consolidate the current understanding of PTDM’s pathogenesis, risk factors, diagnostic challenges, and management strategies, drawing on recent high-quality evidence to inform clinical practice and guide future research.

While previous reviews have comprehensively summarized traditional risk factors and management strategies, they have largely treated metabolic, immunological, and microbial factors as separate entities. In contrast, this review proposes an integrated perspective by positioning gut microbiota dysbiosis as a central mediator linking immunosuppressive therapy to metabolic dysfunction. Furthermore, we incorporate emerging human microbiome data, novel glucose-lowering therapies (SGLT2 inhibitors and GLP-1 receptor agonists), and propose clinically relevant screening and monitoring strategies, aiming to provide a more translational and mechanistic framework for PTDM in the modern transplant era.

We conducted a comprehensive literature search of PubMed, Web of Science, Embase, and Cochrane Library databases through April 2025 using MeSH terms and keywords: “post-transplant diabetes mellitus,” “NODAT,” “kidney transplantation,” “gut microbiota,” “dysbiosis,” and “immunosuppression.” Reference lists were manually reviewed for additional relevant studies.

## 2 Diagnostic criteria and epidemiology

Based on the 2024 International Consensus on Post-Transplantation Diabetes Mellitus (Nephrol Dial Transplant, 2024), the diagnostic framework and screening strategy for PTDM emphasize standardized, OGTT-centered assessment (16).

The oral glucose tolerance test (OGTT) is recommended as the essential tool for both diagnosis and screening and remains the gold standard for detecting PTDM and impaired glucose tolerance (IGT). In contrast, hemoglobin A1c (HbA1c) demonstrates limited diagnostic sensitivity in transplant recipients and should not be used as a standalone diagnostic criterion, particularly in the early post-transplant period, when anemia, fluctuating renal function, and metabolic instability may confound its accuracy (16).

Screening is advised to begin prior to transplantation, ideally while patients are on the waiting list, to enable early identification of high-risk individuals. Following transplantation, an early OGTT (e.g., at 10–13 weeks) is recommended. Importantly, glucose abnormalities detected in the immediate post-operative phase should be interpreted with caution and, where necessary, confirmed at a later time point due to transient metabolic perturbations (16).

Diagnostic classification aligns with established glycemic thresholds: PTDM is defined according to standard diabetes criteria based on fasting plasma glucose and/or 2-hour post-load glucose values derived from OGTT. Prediabetes, encompassing impaired fasting glucose (IFG) and/or IGT, is likewise identified through OGTT and is clinically relevant given its association with increased cardiovascular risk (16).

Given the dynamic metabolic profile of transplant recipients, long-term surveillance is essential. Annual OGTT testing is recommended for individuals with prediabetes or established risk factors for PTDM to facilitate early detection and timely intervention (16–18).

The International Diabetes Federation estimated that the prevalence of diabetes which was 10.5% in 2021, would increase to 11.3% by 2030 and 12.2% by 2040 (19). In contrast, the incidence of PTDM is notably higher and more variable, ranging from 7% to 39% at one year and 10% to 30% at three years post-KT (20–24). This wide variability is due to differences in diagnostic criteria, screening protocols, follow-up duration, pre-transplant patient characteristics, and immunosuppressive regimens (25–27).

## 3 Pathogenesis of PTDM

**Figure 1:**
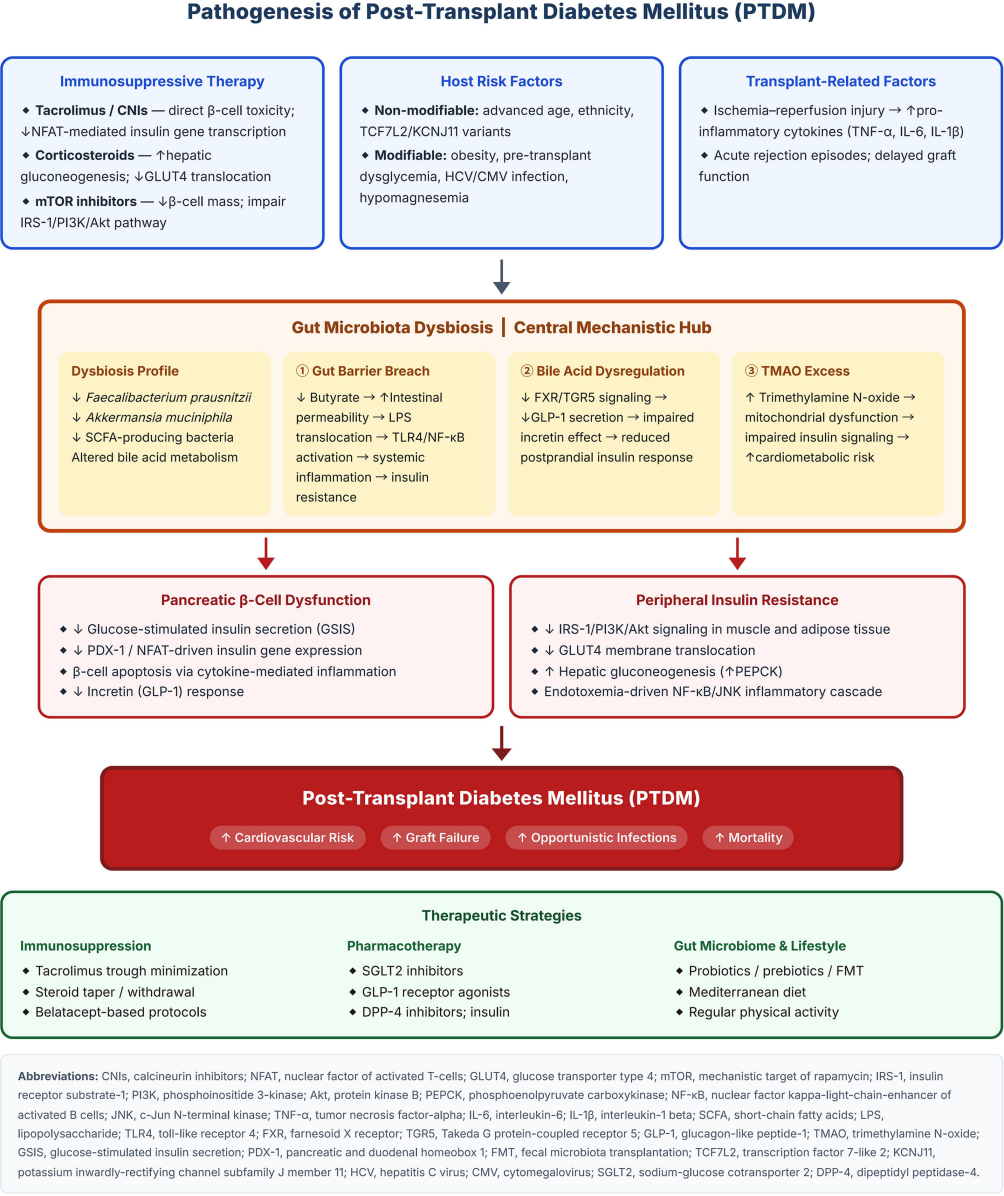
Simplified pathogenesis of post-transplant diabetes mellitus (PTDM). Three upstream contributors — immunosuppressive therapy, host risk factors, and transplant-related inflammatory stimuli — converge on gut microbiota dysbiosis as a central mediator. Dysbiosis promotes PTDM via three principal mechanisms: gut barrier disruption and endotoxemia-driven insulin resistance; impaired bile acid-GLP-1 signaling; and TMAO-mediated mitochondrial dysfunction. These pathways collectively cause pancreatic β-cell dysfunction and peripheral insulin resistance, culminating in PTDM and its major clinical sequelae. Corresponding therapeutic strategies are highlighted at the bottom. CNIs, calcineurin inhibitors; NFAT, nuclear factor of activated T-cells; GLUT4, glucose transporter type 4; mTOR, mechanistic target of rapamycin; IRS-1, insulin receptor substrate-1; PI3K, phosphoinositide 3-kinase; Akt, protein kinase B; PEPCK, phosphoenolpyruvate carboxykinase; NF-kB, nuclear factor kappa-light-chain-enhancer of activated B cells; JNK, c-Jun N-terminal kinase; TNF-a, tumor necrosis factor-alpha; IL-6, interleukin-6; IL-1B, interleukin-1 beta; SCFA, short-chain fatty acids; LPS, lipopolysaccharide; TLR4, toll-like receptor 4; FXR, farnesoid X receptor; TGR5, Takeda G protein-coupled receptor 5; GLP-1, glucagon-like peptide-1; TMAO, trimethylamine N-oxide; GSIS, glucose-stimulated insulin secretion; PDX-1, pancreatic and duodenal homeobox 1; FMT, fecal microbiota transplantation; TCF7L2, transcription factor 7-like 2; KCNJ11, potassium inwardly-rectifying channel subfamily J member 11; HCV, hepatitis C virus; CMV, cytomegalovirus; SGLT2, sodium-glucose cotransporter 2; DPP-4, dipeptidyl peptidase-4.

PTDM is increasingly recognized as a distinct condition from T2DM, despite sharing common pathophysiological pathways (14, 15). Both involve insulin resistance and defective insulin secretion, but PTDM is significantly and uniquely shaped by immunosuppressive drugs, transplant-associated inflammation, and gut microbial disruption. The interplay of these factors is illustrated in **Figure 1**.

### 3.1 Insulin resistance and β-cell secretion defects

At the molecular level, peripheral insulin resistance arises from impaired insulin receptor substrate (IRS-1) phosphorylation and downstream PI3K/Akt signaling in skeletal muscle and adipose tissue, combined with failure to suppress hepatic glucose production via the gluconeogenic pathway (28, 29). β-cell dysfunction in PTDM involves reduced glucose-stimulated insulin secretion (GSIS), impaired mitochondrial oxidative phosphorylation in islet cells, and diminished transcription of insulin gene regulators (30, 31). The enteroinsular axis is further compromised by a reduced incretin effect, with significantly attenuated GLP-1 secretion and action observed in kidney transplant recipients (32, 33). The potential roles of enhanced renal gluconeogenesis and altered central nervous system regulation of metabolism in PTDM warrant further investigation (34–36).

### 3.2 Molecular actions of immunosuppressive agents

Calcineurin Inhibitors (CNIs): Tacrolimus and cyclosporine A inhibit calcineurin, blocking dephosphorylation and nuclear translocation of the Nuclear Factor of Activated T-cells (NFAT)—a transcription factor essential for insulin gene expression and β-cell proliferation (37, 38). *In vitro* studies confirm that prolonged CNI exposure at clinical concentrations suppresses GSIS (39). Tacrolimus additionally exacerbates lipotoxicity through elevation of circulating free fatty acids, creating a synergistic diabetogenic effect that is dose-dependent and partially reversible upon withdrawal (40–42).

Corticosteroids: These agents induce hyperglycemia through multiple molecular mechanisms: (1) upregulation of hepatic phosphoenolpyruvate carboxykinase (PEPCK) driving gluconeogenesis; (2) inhibition of GLUT4 translocation in muscle and adipose tissue; and (3) direct impairment of β-cell insulin secretion via glucocorticoid receptor (GR)-mediated transcriptional suppression (43, 44). Polymorphisms in the GR gene (NR3C1) have been associated with increased PTDM susceptibility (45, 46).

mTOR Inhibitors: Sirolimus and everolimus impair β-cell mass via inhibition of the mTORC1 pathway, leading to reduced β-cell proliferation and induction of apoptosis. Simultaneously, mTOR inhibition disrupts insulin signaling in peripheral tissues by impairing IRS-1-mediated PI3K activation, and exacerbates lipotoxicity (47–50). The diabetogenic risk is amplified when mTOR inhibitors are combined with CNIs (47).

Other Agents: Mycophenolate mofetil does not appear to increase PTDM risk (15). Belatacept (CTLA-4-Ig) demonstrates a more favorable metabolic profile by avoiding CNI-mediated β-cell toxicity (51). Induction therapy with anti-thymocyte globulin or alemtuzumab may confer a lower risk compared to IL-2 receptor antagonists (52, 53).

### 3.3 Inflammatory signaling in PTDM

Chronic low-grade inflammation activates the NF-κB and JNK signaling pathways, leading to elevated circulating concentrations of TNF-α, IL-6, and IL-1β—cytokines that directly impair insulin receptor signaling and promote β-cell apoptosis (54, 55). This inflammatory milieu is further amplified by transplant-related immune activation, creating a molecular environment conducive to glucose dysregulation.

### 3.4 Gut microbiota dysbiosis and the gut-immune-metabolic axis

We propose that alterations in the gut microbiome constitute a central mechanistic node in a “gut-immune-metabolic axis” connecting transplant-related exposures to downstream glucose dysregulation. Kidney transplant recipients exhibit characteristic dysbiosis patterns paralleling those in T2DM, including depletion of SCFA-producing taxa (e.g., *Roseburia*, *Faecalibacterium*) and reduced *Akkermansia muciniphila* abundance (56–59).

The following molecular mechanisms are implicated:

SCFA (butyrate) depletion→compromised intestinal tight junction integrity→ endotoxemia (LPS translocation)→TLR4/NF-κB-driven systemic metabolic inflammation and insulin resistance.
Bile acid dysregulation→impaired FXR/TGR5 signaling→reduced enteroendocrine GLP-1 secretion→attenuated postprandial insulin response.
TMAO accumulation→mitochondrial dysfunction and cardiometabolic risk (58–60).
Immunosuppressant-induced dysbiosis→tacrolimus directly perturbs microbial composition, amplifying β-cell toxicity through a dual-hit mechanism.

Microbiota-targeted therapeutic strategies (prebiotics, probiotics, synbiotics, FMT) remain investigational but are conceptually compelling given this mechanistic framework (60).

## 4 Risk factors for PTDM

**Figure 2:**
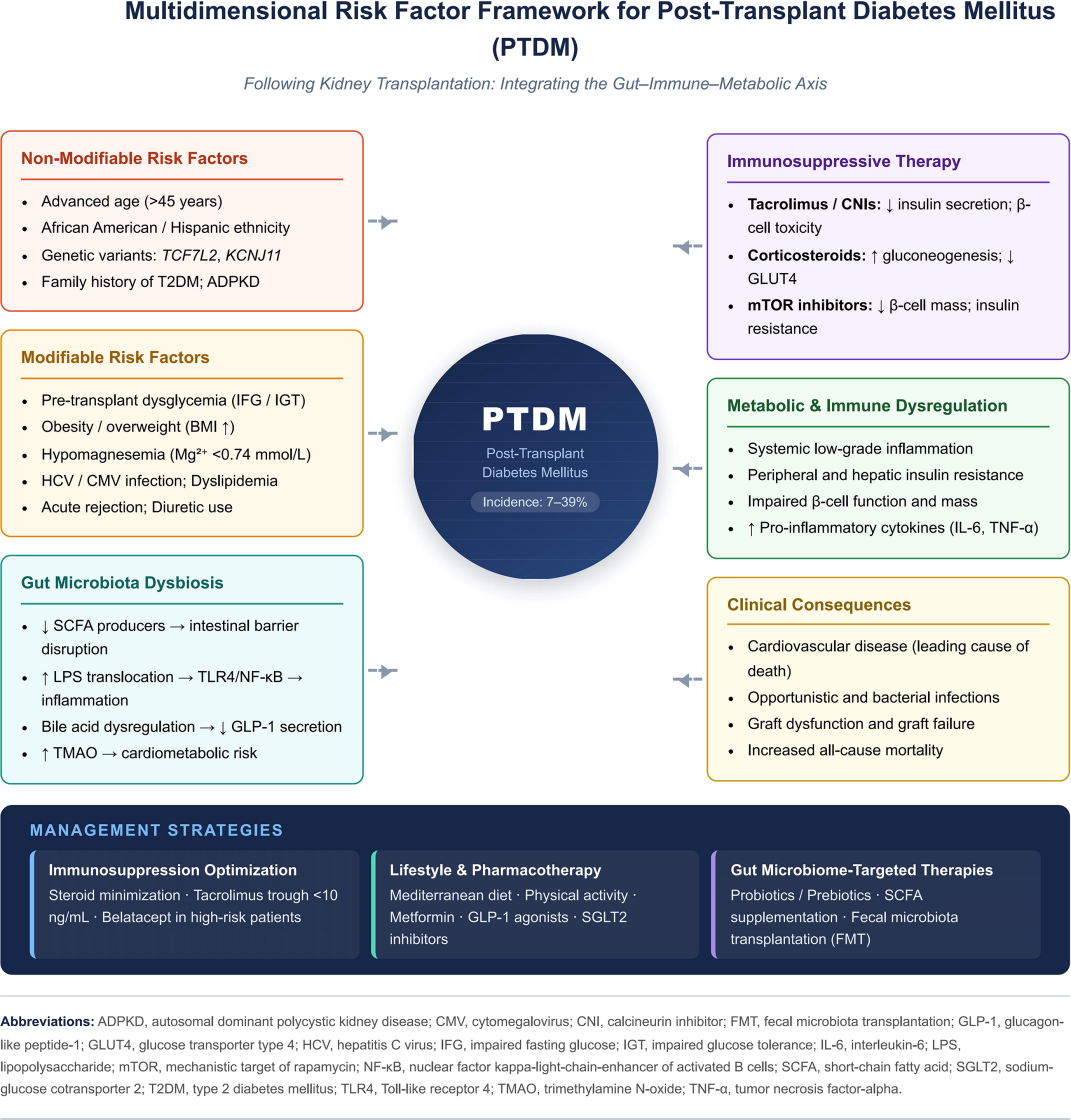
Multidimensional risk factor framework for post-transplant diabetes mellitus (PTDM) following kidney transplantation. Non-modifiable factors (age, ethnicity, genetic predisposition) and modifiable clinical variables (dysglycemia, obesity, viral infections, hypomagnesemia) converge with immunosuppressive therapy to drive gut microbiota dysbiosis and metabolic-immune dysregulation, ultimately precipitating PTDM. Gut microbiota dysbiosis serves as a central mediator, linking immunosuppression-induced perturbations to downstream insulin resistance and β-cell dysfunction. Management requires a multidisciplinary approach integrating immunosuppression optimization, pharmacological and lifestyle interventions, and emerging gut microbiome-targeted strategies. ADPKD, autosomal dominant polycystic kidney disease; CMV, cytomegalovirus; CNI, calcineurin inhibitor; FMT, fecal microbiota transplantation; GLP-1, glucagon-like peptide-1; GLUT4, glucose transporter type 4; HCV, hepatitis C virus; IFG, impaired fasting glucose; IGT, impaired glucose tolerance; IL-6, interleukin-6; LPS, lipopolysaccharide; mTOR, mechanistic target of rapamycin; NF-kB, nuclear factor kappa-light-chain-enhancer of activated B cells; SCFA, short-chain fatty acid; SGLT2, sodium-glucose cotransporter 2; T2DM, type 2 diabetes mellitus; TLR4, Toll-like receptor 4; TMAO, trimethylamine N-oxide; TNF-a, tumor necrosis factor-alpha.

Risk factors for PTDM are broadly classified into non-modifiable (intrinsic patient characteristics) and modifiable (clinical and environmental) categories. This section focuses exclusively on clinical associations and epidemiological evidence. **Figure 2** illustrates the Multidimensional Risk Factor Framework for PTDM.

### 4.1 Non-modifiable risk factors

Age: Advanced age is an independent risk factor for PTDM (20, 61, 62). Recipients over 45 years of age face more than a two-fold increased risk compared to younger patients (63–65), consistent with the age-related decline in β-cell reserve observed in the general population (66, 67).

Race and Ethnicity: African American and Hispanic recipients have a markedly higher risk of PTDM compared to Caucasian recipients (64, 68). South Asian populations also exhibit a significantly elevated incidence (69). These disparities reflect higher background prevalence of T2DM and ethnic differences in genetic susceptibility (70, 71).

Genetic Predisposition: A family history of diabetes increases PTDM risk. The most robust genetic association is with the TCF7L2 variant rs7903146 (72, 73). Other implicated loci include KCNJ11, PPARG, and HNF1B (74–76).

Autosomal Dominant Polycystic Kidney Disease (ADPKD): Multiple studies and meta-analyses identify ADPKD as an independent risk factor for PTDM (77–81), possibly mediated in part by specific HLA subtypes (e.g., HLA-A28, HLA-B13) (82).

### 4.2 Modifiable risk factors

Obesity and BMI: Pre-transplant obesity is a well-established risk factor (83, 84). A large multicenter study demonstrated that each 5 kg/m² increase in BMI was associated with an adjusted hazard ratio of 1.19 for PTDM (61). Post-transplant weight gain has also been associated with PTDM development (85, 86).

Pre-Transplant Dysglycemia: Impaired fasting glucose (IFG) and impaired glucose tolerance (IGT) are strong independent predictors of PTDM (87–89).** A** retrospective study of 597 non-diabetic kidney transplant recipients found that pre-transplant IGT conferred a 3.8-fold increased risk—a stronger predictor than age alone (90). The KDIGO 2020 guideline recommends pre-transplant OGTT for all candidates (91).

Hypomagnesemia: Low serum magnesium, commonly induced by CNI use, is independently associated with PTDM (92–94). For every 0.1 mmol/L decrease in serum magnesium, the risk of PTDM increases by approximately 20% (95, 96).

Viral Infections:

HCV: Hepatitis C infection increases PTDM risk by 30–40% (97–99), independent of immunosuppression (100, 101).
CMV: Both symptomatic and asymptomatic CMV infection are associated with increased PTDM risk. Asymptomatic CMV infection has been linked to a four-fold increased risk (102, 103).

Transplant-Related Clinical Factors:

Acute Rejection: Episodes of acute rejection are independently associated with increased PTDM incidence (104, 105), partly attributable to high-dose corticosteroid treatment required for rejection management.
Delayed Graft Function (DGF): The association between DGF and PTDM remains inconsistent across studies. While some studies identify DGF as an independent risk factor (106), others report no significant correlation (27). The observed heterogeneity likely reflects residual confounding arising from differences in ischemia–reperfusion injury severity, variability in corticosteroid exposure during DGF management, and inconsistent definitions of DGF across study populations. In light of the limited and conflicting evidence, DGF should be considered a putative—rather than definitive—independent risk factor for PTDM. Well-designed prospective studies employing standardized DGF criteria are therefore needed to clarify this association.

Other Modifiable Factors:

Diuretic Use: A prospective cohort study found a 3.28-fold increased hazard for PTDM associated with diuretic use (107).
Dyslipidemia: Elevated remnant lipoprotein (RLP) cholesterol is a novel, independent predictor of PTDM (108).
Metabolic Syndrome: Pre-transplant metabolic syndrome significantly increases PTDM risk (109, 110).
Visceral Adiposity: Visceral adipose tissue volume correlates more strongly with PTDM than BMI alone (111).
HLA Mismatch: Evidence remains conflicting (61, 112).

## 5 Diagnosis and challenges

Accurate diagnosis of PTDM is essential but presents several challenges. should be followed with the following key considerations:

(1) Distinction from Pre-existing Diabetes: Pre-transplant diabetes screening practices vary across centers, with many relying solely on FPG and HbA1c, both of which are less sensitive than OGTT in CKD/ESRD patients (113). Consequently, some cases diagnosed as PTDM may in fact represent undiagnosed pre-transplant diabetes.
(2) Transient Post-Transplant Hyperglycemia: Hyperglycemia is common immediately following transplantation due to surgical stress, high-dose corticosteroids, and other immunosuppressive agents. This “transient hyperglycemia” typically resolves as doses are reduced and clinical status stabilizes. Consensus guidelines recommend postponing the formal diagnosis of PTDM until the patient is on a stable, maintenance immunosuppressive regimen and renal function has recovered (9, 114).
(3) Limitations of HbA1c: As noted earlier, HbA1c can be unreliable during the first year post-transplant. Although an HbA1c ≥ 6.5% may be used for diagnosis, it should not be the sole screening tool during this period (9, 18).

## 6 Prevention and management strategies

### 6.1 Modification of immunosuppression

Given that immunosuppressants account for up to 74% of the attributable risk for PTDM, modifying the immunosuppressive regimen is a primary strategy (27).

Corticosteroids: Early and rapid tapering of corticosteroids to a low maintenance dose (e.g., ≤5 mg/day prednisone) or the use of steroid-free regimens can significantly reduce the risk of PTDM (115).

CNIs: Minimizing tacrolimus exposure by maintaining target trough levels below 10 ng/mL effectively reduces the incidence of PTDM (27). In high-risk patients, conversion from tacrolimus to cyclosporine or a CNI-free regimen (e.g., using belatacept) may be considered, balancing the risks of rejection and metabolic complications (51, 116).

mTOR Inhibitors: Given their potent diabetogenic effect, particularly when combined with CNIs, careful consideration is required when using sirolimus or everolimus in high-risk patients (47).

### 6.2 Lifestyle and pharmacological interventions

Lifestyle Modifications: Pre- and post-transplant weight loss programs, dietary counseling (e.g., Mediterranean diet, reduced sugar intake), and regular physical activity are essential for improving insulin sensitivity and preventing PTDM (117).

Pharmacotherapy: Metformin is often the first-line therapy but requires careful monitoring of renal function. GLP-1 receptor agonists and DPP-4 inhibitors provide effective glycemic control with a low risk of hypoglycemia and may be suitable options (118). Insulin therapy is often required, particularly in the early stages.

In addition to traditional agents, newer antidiabetic drugs are reshaping PTDM management. SGLT2 Inhibitors: Improve glycemic control independently of insulin Provide cardiorenal protection Emerging evidence suggests safety in kidney transplant recipients, although infection risk requires monitoring (119–121). GLP-1 Receptor Agonists: Promote weight loss and improve insulin sensitivity Restore incretin effect May counteract immunosuppression-induced metabolic dysfunction (122, 123).

These agents represent a paradigm shift and are not adequately addressed in earlier PTDM reviews.

### 6.3 Management of specific risk factors

Hypomagnesemia: While oral magnesium supplementation improves insulin sensitivity in the general diabetic population, its efficacy in preventing PTDM in KTRs remains unconfirmed and requires further investigation (124).

Viral Infections: Pre-emptive or prophylactic antiviral therapy for CMV and successful eradication of HCV with direct-acting antivirals are recommended and may reduce the associated risk of diabetes (97, 102).

Other Measures: Judicious use of diuretics, management of dyslipidemia, and optimal HLA matching (where possible) are additional considerations.

### 6.4 Targeting the gut microbiome

Modulating the gut microbiome presents a promising frontier. Interventions such as prebiotics (dietary fibers), probiotics, and postbiotics (e.g., SCFA supplements) aimed at restoring a healthy microbial balance and enhancing SCFA production may potentially alleviate glucose intolerance in KTRs (60, 125). However, robust clinical trials are required before these strategies can be incorporated into standard practice.Future strategies may include precision microbiome modulation tailored to individual metabolic profiles.

## 7 Conclusion and future directions

PTDM remains a major barrier to long-term transplant success.This review advances the field by proposing a unifying “gut–immune–metabolic axis” and a multi-hit model integrating immunosuppressive therapy, microbiota dysbiosis, and host metabolic susceptibility. By incorporating emerging therapies (SGLT2 inhibitors, GLP-1 receptor agonists), donor-related factors, and AI-driven risk prediction, we provide a more comprehensive and clinically relevant framework than prior reviews.

## 8 Future directions should prioritize

Microbiome-based interventions.

Precision immunosuppression.

AI-integrated risk prediction models.

Ultimately, a shift toward personalized, mechanism-based management may significantly reduce the burden of PTDM and improve graft and patient outcomes.
